# Kaempferol Attenuates ROS-Induced Hemolysis and the Molecular Mechanism of Its Induction of Apoptosis on Bladder Cancer

**DOI:** 10.3390/molecules23102592

**Published:** 2018-10-10

**Authors:** Ping Wu, Xiaofeng Meng, Huade Zheng, Qin Zeng, Tianfeng Chen, Wen Wang, Xia Zhang, Jianyu Su

**Affiliations:** 1 School of Food Science and Engineering, South China University of Technology, Guangzhou 510640, China; pingwuscut@gmail.com (P.W.); 18700933573@163.com (X.M.); wangwen20139146@163.com (W.W.); cexzhang@scut.edu.cn (X.Z.); 2 Guangdong Province Key Laboratory for Green Processing of Natural Products and Product Safety, Guangzhou 510640, China; 3 Department of Materials Science and Engineering, South China University of Technology, Guangzhou 510640, China; 4 Fischell Department of Bioengineering, University of Maryland, College Park, MD 20742, USA; qzeng8156@gmail.com; 5 Department of Chemistry, Jinan University, Guangzhou 510632, China; tchentf@jnu.edu.cn

**Keywords:** kaempferol, antioxidant activity, anti-bladder cancer cell activity, apoptosis, p53 pathway

## Abstract

Bladder cancer has become the most common malignant urinary carcinoma. Studies have shown that significant antioxidant and bladder cancer-fighting properties of several plant-based diets like *Psidium guajava*, ginger and amomum, are associated with their high kaempferol content. In this paper, we evaluated the antioxidant and anticancer activities of kaempferol and its mechanism of induction to apoptosis on bladder cancer cells. Our findings demonstrated that kaempferol showed an obvious radical scavenging activity in erythrocytes damaged by oxygen. Kaempferol promoted antioxidant enzymes, inhibited ROS generation and lipid peroxidation and finally prevented the occurrence of hemolysis. Additionally, kaempferol exhibited a strong inhibitory effect on bladder cancer cells and high safety on normal bladder cells. At the molecular level, kaempferol suppressed EJ bladder cancer cell proliferation by inhibiting the function of phosphorylated AKT (p-AKT), CyclinD1, CDK4, Bid, Mcl-1 and Bcl-xL, and promoting p-BRCA1, p-ATM, p53, p21, p38, Bax and Bid expression, and finally triggering apoptosis and S phase arrest. We found that Kaempferol exhibited strong anti-oxidant activity on erythrocyte and inhibitory effects on the growth of cancerous bladder cells through inducing apoptosis and S phase arrest. These findings suggested that kaempferol might be regarded as a bioactive food ingredient to prevent oxidative damage and treat bladder cancer.

## 1. Introduction

Nowadays, bladder cancer has become the most common malignant urinary carcinoma because people are easily exposed to many carcinogens including tobacco and odorous chemical fumes [1]. Moreover, bladder cancer is more vulnerable to dispersal or metastasis throughout the body and to relapse. These factors make bladder cancer more difficult to cure. Generally, the most effective methods to treat most cancers involve chemotherapeutic drugs, including doxorubicin, hydroxyl-camptothecin and mitomycin [2]. However, the bladder cancer cells display insensitivity to these drugs due to their general drug resistance and acidic environment. Therefore, finding compounds with high antioxidant and antitumor activities from plant-derived foods becomes the focus of research attention.

**Figure 1:**
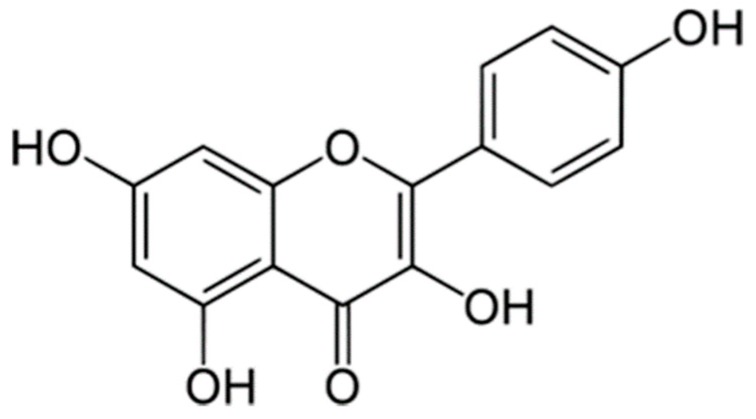
Chemical structure of kaempferol.

Kaempferol (Figure 1) is a flavonoid abundantly found in *Psidium guajava*, ginger, propolis and teas [3,4,5]. The anti-inflammation and anti-diabetic activity of kaempferol has gained much attention [6,7]. Studies also demonstrated that kaempferol could obviously protect the cell viability from ROS, which would cause extensive oxidative damage by oxidizing macromolecules such as DNA, protein, and lipid. In addition, evidence has shown that the application of kaempferol could suppress the risk of developing tumors, such as pancreatic cancer, gastric cancer and lung cancer [8,9]. Moreover, researchers have discovered that kaempferol showed antiproliferation and induced apoptosis of bladder cancer cells such as 5637 and T24 cells [10]. All these findings highlight that kaempferol would be a potential chemotherapeutic agent to treat bladder cancer. However, abundant underlying mechanisms of apoptosis and cell cycle arrest of kaempferol in bladder cancer EJ cells have not been declared.

In this work, we study the intracellular antioxidant oxidative stress and anti-hemolysis of kaempferol in human erythrocytes. Since intracellular oxidative damage was closely involved in the tumorigenesis, we investigated the kaempferol-induced antiproliferative effect and the molecular mechanisms of apoptosis and cell cycle arrest in bladder cancer EJ cells. Collectively, our results discovered that kaempferol could inhibit hemolysis by mediating intracellular antioxidant enzymes. Kaempferol induced apoptosis and S phase arrest effect bladder cancer cells by inhibiting the function of phosphorylated AKT (p-AKT), CyclinD1, CDK4, Bid, Mcl-1 and Bcl-xL and promoting p-BRCA1, p-ATM, p53, p21, p38, Bax and Bid expression. This study suggests that kaempferol would be a therapeutic agent to treat bladder cancer.

## 2. Results

### 2.1. Kaempferol Suppresses 2,20-azobis (2-amidinopropane)(AAPH)-Induced Oxidative Damage in Human Erythrocytes

AAPH could initiate water-soluble free radicals to induce radicals such as ROO**·**, and cause DNA peroxidation and erythrocyte hemolysis through attacking the lipids and proteins of cell membranes [11]. We set out to examine the protective effects of kaempferol on erythrocytes from AAPH-induced oxidative damage.

As shown in Table 1, the hemolysis inhibition rate was 42.15 ± 0.0025% when erythrocytes were only treated with 200 mM AAPH. However, co-treatment of the erythrocytes with kaempferol could improve hemolysis inhibition rate in a dose-dependent manner. The results demonstrated that 80 μM kaempferol exhibited nearly equal protective effects of vitamin C at the same concentration on the AAPH-induced erythrocyte hemolysis (*p* > 0.05).

**Figure 2:**
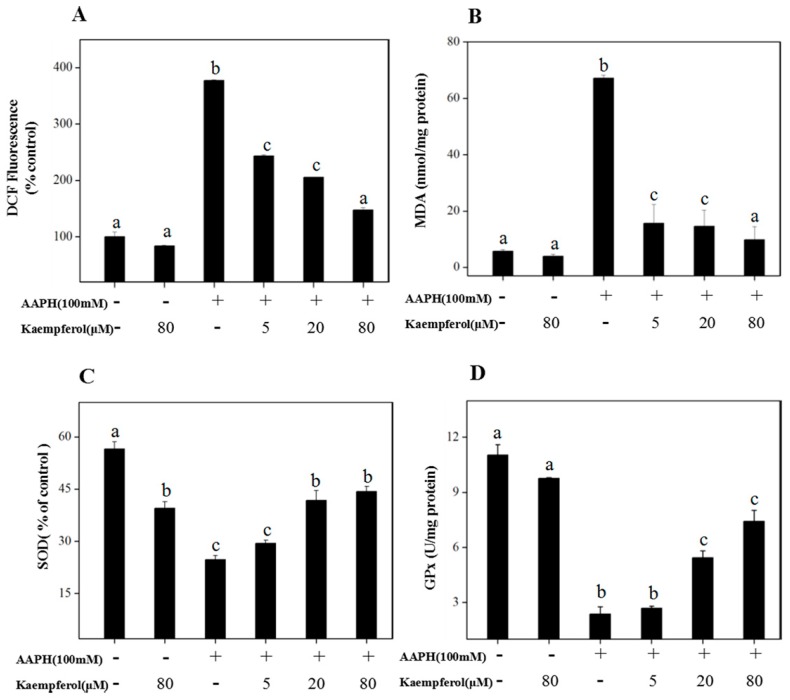
Protection of kaempferol against AAPH-induced oxidative damage. (**A**) Kaempferol inhibited AAPH-induced ROS generation in erythrocytes. (**B**) Kaempferol attenuated AAPH-induced MDA generation in erythrocytes. (**C**) Kaempferol enhanced AAPH-induced SOD activity in erythrocytes. (**D**) Kaempferol enhanced AAPH-induced GPx activity in erythrocytes. All data are expressed as means ± SD of triplicates. Means with different letters (a, b, c) are statistically different at *p* < 0.05 level.

To further investigate the protective effects of kaempferol on erythrocytes from AAPH-induced oxidative injury, 2′,7′-dichlorofluorescin diacetate (DCF) dye was applied to determine intracellular ROS. As shown in Figure 2A, intracellular ROS production, which was 377.3% at the control, markedly increased when erythrocytes were treated with 200 mM AAPH for 2 h. However, when erythrocytes were pretreated with 5, 20 and 80 μM kaempferol, intracellular ROS experienced a significant drop to 243.4%, 204.6% and 147.3%, respectively. Moreover, the absence of significant differences between 80 μM kaempferol alone and the control (*p* > 0.05) indicated that kaempferol had no obvious cytotoxicity towards normal erythrocytes.

As shown in Figure 2B, the content of MDA in the erythrocytes obviously increased to 67.10 nmol/mg protein when erythrocytes were treated with 200 mM AAPH alone for 2 h. However, this effect was remarkably reversed by pretreating with kaempferol, dependent on the concentration. As displayed in Figure 2C,D, kaempferol did increase the content of superoxide dismutase (SOD) and GPx in erythrocytes, which might help erythrocytes avoid AAPH-induced oxidative damage. SOD activity was up to 41.79% of control when erythrocytes were exposed to 20 μM kaempferol in advance, and the SOD activity dropped to 24.70% of control when erythrocytes were treated with 200 mM AAPH alone. Abundant studies have already shown that the antioxidancy of flavonoids is associated with their influence on intracellular antioxidant enzymes including SOD and GPx. Our experiments showed similar results as in Figure 2D. The activity of GPx was 7.41 U/mg protein when erythrocytes were pretreated with 80 μM kaempferol for 20 min, while the GPx activity decreased to 2.37 U/mg protein when exposed to 200 mM AAPH.

**Figure 3:**
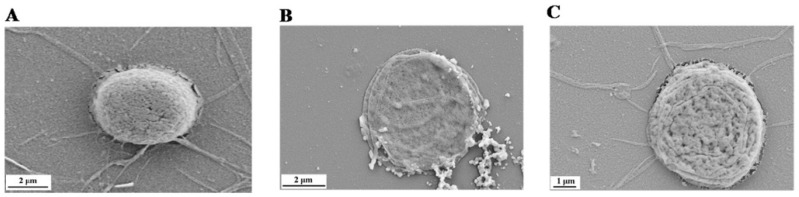
Protective effects of kaempferol on the images of human erythrocytes by scanning electron microscopy. (**A**) Normal human erythrocytes. (**B**) Damaged erythrocytes by 100 mM AAPH alone for 2 h. (**C**) Pretreated erythrocytes with 80 μM kaempferol before erythrocytes were cultured with 100 mM AAPH for 2 h.

To further investigate the protective effects of kaempferol from ROS-induced hemolysis, the morphologic changes of human erythrocytes were obtained by SEM. Normal human erythrocytes as controls are shown in Figure 3A, displaying the tridimensional state with typical arched shape. When erythrocytes were treated with 200 mM AAPH for 2 h, it resulted in cell collapse and structure failure as shown in Figure 3B. However, 80 μM kaempferol could neutralize the AAPH perturbing effect and restore the three-dimension structure (Figure 3C). This intuitive observation confirmed that kaempferol could protect erythrocytes against AAPH-induced oxidative damage.

### 2.2. Antiproliferative Effect of Kaempferol on Bladder Cancer Cells

**Figure 4:**
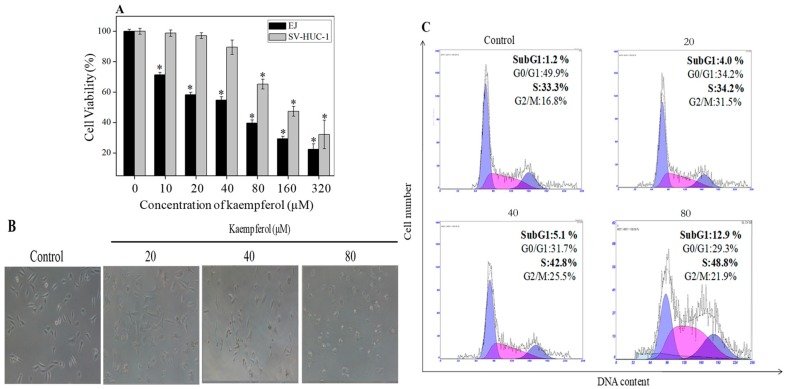
Protective effects of kaempferol on bladder cancer EJ cells. (**A**) The effects of kaempferol on the cell viability of an EJ cell and a normal bladder cell SV-HUC-1. (**B**) Morphological changes of EJ cells as examined by phase-contrast microscopy (magnification, 200×). (**C**) Kaempferol-induced S cell cycle arrest in EJ cells. Cell cycle distribution of EJ cells treated with kaempferol was analyzed by flow cytometric analysis. ∗ *p* < 0.05 versus the control.

To demonstrate the antiproliferative effect of kaempferol on bladder cancer cells, MTT assay was performed. As displayed in Figure 4A, the viability of bladder cancer EJ cells decreased in a dose-dependent manner when treated with concentrations ranging from 10 to 320 μM. For instance, EJ cell viability decreased from 58.26% to 50% when treated with kaempferol from 20 μM to 54.7 μM. In addition, our results showed that there was no significant effect on the growth of normal bladder cell SV-HUC-1 when treated with kaempferol from 10 to 40 μM. Nevertheless, kaempferol with concentration as high as 80 μM demonstrated cytotoxicity to SV-HUC-1. Furthermore, the effect of kaempferol on EJ cell morphology was further confirmed by phase-contrast microscopy observation. Figure 4B showed that morphological changes on EJ cells were attenuated by different concentrations of kaempferol. Moreover, the numbers of EJ cells decreased gradually and the cells became rough with shrinkage compared to control cells, with the increasing concentrations of kaempferol. These results were also consistent with our investigation of EJ cell viability.

To further clarify how kaempferol inhibits EJ cells proliferation, flow cytometric analysis was carried out to observe the cell cycle distribution. As illustrated in Figure 4C, the SubG1 phase and S phase of EJ cells dramatically increased when EJ cells were treated with increasing concentration of kaempferol. So, the SubG1 proportion increased from 1.2% (control) to 4.0%, 5.1% and 12.9% at concentration of 20 μM, 40 μM and 80 μM kaempferol, respectively. Additionally, there was an increase in the S phase from 33.3% (control) to 34.2%, 42.8% and 48.8%, respectively. It can be concluded that kaempferol inhibited EJ cells growth mainly through induction of apoptosis and S cell cycle arrest.

### 2.3. Molecular Mechanism of Kaempferol-Induced Apoptosis and S Phase Arrest in EJ Cells

**Figure 5:**
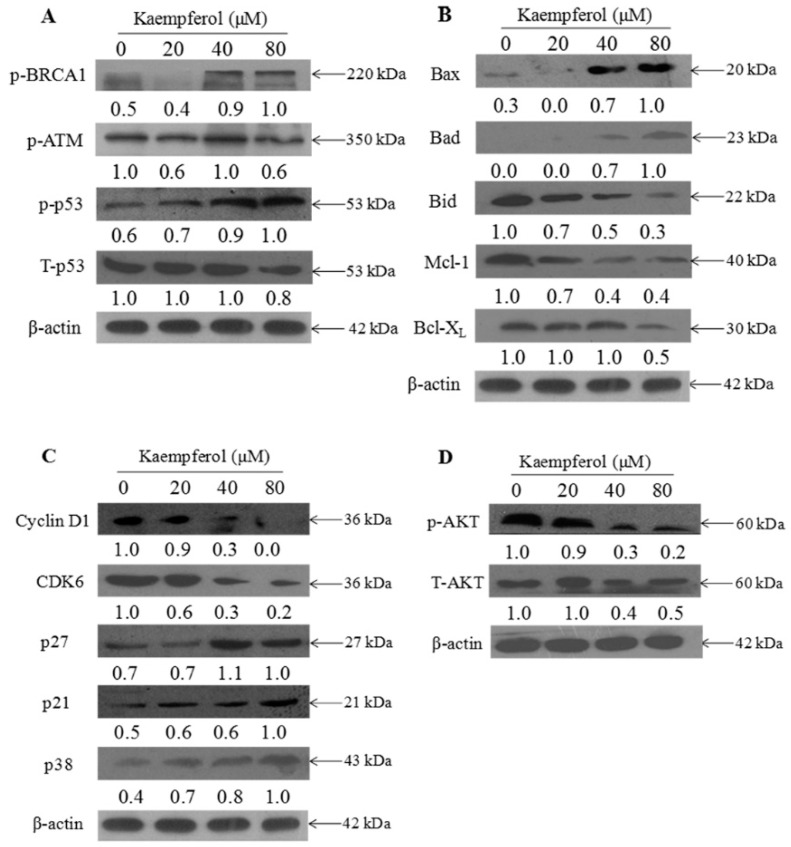
The expression levels of signal protein in EJ cells treated with kaempferol by western blotting. (**A**) p-BRCA1, p-ATM, p-p53, and T-p53. (**B**) Bax, Bad, Bid, Mcl-1, and Bcl-xL. (**C**) Cyclin D1, CDK6, p27, p21, and p38. (**D**) Phosphorylated AKT (p-AKT) and total AKT (t-AKT). β-actin was used as the loading control. The protein expression levels are percentages of the control. Relative intensity was shown above each immunoblotted protein and condition. Representative images of three independent experiments are presented.

To further confirm the underlying molecular mechanism to clarify the kaempferol-induced apoptosis, western blotting was employed to detect the expression of p53, ATM and Bcl-X2 family. As shown in Figure 5A, when EJ cells were treated with kaempferol varying from 20 μM to 80 μM., the expression levels of p-BRCA-1 and p-p53 increased, while the expression level of total p53 (T-p53) slightly decreased compared to the negative control. Meanwhile, kaempferol down-regulated the expression of anti-apoptotic proteins (Bid, Mcl-2, and Bcl-xL), while up-regulated the pro-apoptotic proteins (Bax and Bad) in EJ cells, as shown in Figure 5B.

On the basis of an observed S cycle arrest, we also examined the expression level of p-AKT. As displayed in Figure 5C, S-related proteins (CyclinD1 and CDK6) showed a noticeable down-regulation in EJ cells when treated with increasing dose of kaempferol. In addition, p21, p27, and p38 exhibited an upward trend, leading to the inhibition of Cyclin D1 and CDK6, which induced S phase arrest. Figure 5D illustrates that EJ cells exposed to kaempferol did not generate any change in the expression level of total AKT (t-AKT), but the p-AKT level decreased in a dose-dependent manner.

## 3. Discussion

Heme-containing proteins promote oxidative processes and make human erythrocytes susceptible to peroxidative damage. The membrane is an oxygen-rich environment with hemoglobin that is iron-rich [12,13]. The lipid bilayer structure of erythrocyte is critical to the cytoskeletal network organization with the red blood cells. Increasing evidence has demonstrated that excessive free radicals such as O_2_**·**, H_2_O_2_ and NO might damage the lipid bilayer structure of erythrocyte and induce several diseases including atherosclerosis, hypertension, inflammatory arthritis and diabetes [14,15]. Meanwhile, ROS initiates lipid peroxidation reactions. All of these together could cause the membrane integrity damage and even cell death [16,17]. MDA is the final product of membrane lipid peroxidation. Accumulating MDA might lead to the impairment of membrane-related functions and diminish erythrocyte survival [18,19]. In this investigation, 20 μM kaempferol displayed obvious protective effects on erythrocytes against AAPH-induced oxidative damage (Table 1). This might partially be ascribed to kaempferol-induced increased activities of antioxidant enzymes (GPx) and decreased MDA content, which would directly scavenge ROS in oxidative erythrocytes (Figure 2). These results were consistent with previous research, showing kaempferol could mediate the activities of antioxidant and pro-oxidant enzymes [20]. Furthermore, some reports have shown that kaempferol induced energetic failure by inhibiting both Complex I and glucose uptake of the mitochondrial respiratory chain in HeLa cells [21]. In addition, kaempferol could regulate mitochondrial function through preventing mitochondrial membrane potential dissipation, complex IV inactivation, and ROS production in MC3T3-E1 cells treated with antimycin A [22]. All these actions suggested that kaempferol could remarkably attenuate oxidative stress. Unexpectedly, we discovered that the SOD content was reduced in erythrocyte treated with 80 μM kaempferol compared to the control. The phenomena suggest that high doses of kaempferol may cause some potential side-effects. Similar studies conducted by Vellosa et al. discovered that while pretreatment with kaempferol does could reduce AAPH effects, kaempferol alone could cause even stronger hemolysis inhibition rate than AAPH [23]. Therefore, it is very important to make decisions about the most effective dose of kaempferol to avoid additional adverse effects. Further research would be needed to evaluate the safe dosage for clinical trials.

Studies have demonstrated that intracellular antioxidant oxidative stress had a direct association with the occurrence of cancer [24]. Kaempferol shows remarkable intracellular antioxidant ability, which may lead to the growth inhibition effect on cancer cells. In the present study, kaempferol exhibited strong antiproliferative effects on EJ cells. These results were consistent with the recent research results [10,25]. The antiproliferative activity of cancer cells was related to the induction of apoptosis. Our results of flow cytometric analysis displayed that kaempferol obviously inhibited EJ cell growth mainly through induction of S cell cycle arrest and apoptosis. CDK6 exhibits a vital role in the G0/1-S transition through modulating cyclin D [26]. It has become a desirable target in cancer therapies [27]. Our mechanistic investigations indicated that kaempferol obviously down-regulated the expression level of CyclinD1 and CDK6, and up-regulated the expression level of p21, p27 and p38 which would induce to the inhibition of Cyclin D1 and CDK6 (Figure 5). These results were consistent with the flow cytometric analysis. Some similar results were declared in a research elucidating that kaempferol affected the cell cycle of EJ cells by up-regulated p21 waf1/cip1.

P53, a tumor suppressor protein, played a significant role in the processes of apoptosis and S cell cycle arrest. It could indirectly or directly up-regulate pro-apoptotic proteins such as Bax, and down-regulate anti-apoptotic proteins such as Bcl-2 [28]. Besides, p53 could activate p21, a multifunctional tumor suppressor, which would induce apoptosis and cell growth arrest [29]. In this study, EJ cells exposed to kaempferol resulted in up-regulated expression level of phosphorylated status of p53 (p-p53). The p-p53 could affect mitochondria-mediated apoptotic signaling pathways characterized by up-regulated Bax and Bad and down-regulated Bid, Mcl-1, and Bcl-xL (Figure 5). Moreover, the enhancement of p53 could increase the expression level of p21, p27 and p38, which were members of the CDK inhibitors related to S cell cycle arrest [30]. In addition, kaempferol increased expression level of p-ATM and p-BRCA1, which would induce DNA damage. 

Additionally, we examined the expression level of t-AKT and p-AKT. AKT signal pathway showed a vital role in regulating cell progression and inhibiting the pro-apoptotic protein expression [31]. It was reported that most of bladder cancers exhibited obviously higher p-AKT levels compared to control groups. Therefore, AKT is the switching locus for the cancer phenotype [32]. In the present study, we found that kaempferol decreased the p-AKT level, while the t-AKT level did not change in EJ cells. These results were consistent with previous research [33]. Therefore, kaempferol is an inducer of EJ cells apoptosis. While the bioavailability of kaempferol is relatively low due to first-pass metabolism in the gut and liver [34], evidence has demonstrated that kaempferol could exhibit effective antineoplastic potential in bladder cancer by actions in both blood and urine [35]. Moreover, Qiang Dang et al. pointed out that kaempferol could obviously inhibit the growth of bladder cancer tumor through inducing apoptosis [10]. All these findings indicated that kaempferol would be an effective herbal medicine to use as a novel chemotherapeutic drug to treat bladder cancer.

## 4. Materials and Methods 

### 4.1. Materials

Kaempferol, ascorbic acid (vitamin C), AAPH, MTT and PI were obtained from Sigma-Aldrich (Sigma-Aldrich, St. Louis, MO, USA). ROS assay kits, GPx assay kits and SOD assay kits were purchased from Beyotime Institute of Biotechnology (Shanghai, China). BCA protein and MDA kits were purchased from Nan Jing Jian Cheng Bioengineering Institute (Nanjing, China). All antibodies were obtained from Cell Signaling Technology (Beverly, MA, USA). The antibiotic mixture (penicillin−streptomycin), Dulbecco’s modified Eagle’s medium (DMEM), and fetal bovine serum (FBS) were purchased from Invitrogen (Carlsbad, CA, USA). The human bladder cancer EJ cells and normal bladder cells SV-HUC-1 were purchased from American Type Culture Collection (ATCC; Manassas, VA, USA).

### 4.2. Erythrocyte Hemolytic Assay Mediated by AAPH

The method was carried out according to Zhao et al. [36]. Briefly, fresh blood including heparin from a healthy volunteer was collected and centrifuged at 1500 g for 10 min. A 20% erythrocytes suspension was obtained after erythrocytes were washed and suspended in phosphate buffer saline (PBS, pH 7.4). Different concentrations of kaempferol were added to 0.1 mL obtained erythrocytes suspension, and were then incubated at 37 °C for 2 h. Then we added 0.2 mL of 200 mM AAPH and incubated at 37 °C for 2 h. Then, 0.3 mL of PBS and Milli-Q water were co-incubated with 20% erythrocytes as a hemolytic and completely hemolytic control, respectively. After incubation, the mixture was centrifuged at 1500 g for 10 min. The absorbance of supernatant was determined at 540 nm by microplate reader. Ascorbic acid (80 μM) was used as a positive control.

### 4.3. Scanning Electron Microscope (SEM) Observation

A 10 μL treated erythrocytes suspension was dropped on the silicon pellet. Then 2.5% glutaraldehyde was applied to fix erythrocytes for 5 min [37]. After the cells washed with PBS, its morphology was obtained under SEM (Jena, Carl Zeiss AG, Merlin, Germany).

### 4.4. Determination of ROS Generation, MDA Content, and Enzyme Activities of SOD and Gpx

Intracellular ROS, MDA, GPx and SOD content were detected according to Zhao et al. [36]. The intracellular ROS of the collected erythrocytes was examined by DCF assay. Briefly, 10 μM DCFH-DA was applied to the treated erythrocytes for 20 min (37 °C). The cells were added to 96-well plates for incubation after being washed by PBS. The fluorescence intensity was examined by a fluorescence reader (Thermo Fisher Variosban Flash, Waltham, MA, USA) (Ex = 488 nm, Em = 525 nm). The MDA contents and determination of enzyme activities of GPx and SOD were measured according to instructions of the corresponding assay kits.

### 4.5. Culture and Cell Viability Assay

EJ and normal bladder SV-HUC-1 cells were grown in Dulbecco’s modified Eagle’s medium (DMEM) supplemented with 10% fetal bovine serum (FBS), 100 units/mL penicillin and 50 units/mL streptomycin in a humidified atmosphere at 37 °C with 5% CO_2_. These two types of cells viabilities were quantified by MTT assay [38]. The EJ cells and SV-HUC-1 cells were seeded at 2 × 10^4^ cells/well and 5 × 10^4^ cells/well in 96-well culture plates overnight, respectively. After pretreated with different concentrations of kaempferol for 72 h, 30 μL of 5 mg/mL MTT was added and incubated for another 4 h. The crystals were dissolved in 150 μL/well of DMSO following that the supernatant fluid was deprived. A microplate reader was applied to examine absorbance with wavelength at 570 nm.

### 4.6. Flow Cytometric Analysis

Flow cytometric analysis was applied to detect cell cycle distribution reported previously [39]. Briefly, bladder cancer EJ cells were treated with 20, 40, and 80 μM kaempferol for 48 h, respectively. Then EJ cells were fixed with 70% cold ethanol after being washed with PBS. A flow cytometer (Beckman Coulte, Miami, FL, USA.) was applied to determine the stained cells which were stained with PI (a fluorescent intercalating agent that can be used to stain cells) for 30 min in darkness. 

### 4.7. Western Blotting Analysis

The cellular proteins of EJ cells were determined by western blotting [40]. Briefly, the proteins were harvested after cells lysed with radioimmunoprecipitation assay (RIPA) buffer and centrifugated at 12,000 rpm (4 °C). After proteins were quantified with BCA kit, protein samples were separated via sodium dodecyl sulfate-polyacrylamide gel electrophoresis (SDS-PAGE) and transferred to polyvinylidene fluoride (PVDF) membranes. The membranes were incubated with primary antibodies after being blocked in 5% nonfat milk. Then they were incubated with secondary antibodies and finally detected with X-ray films. The densities the bands were calculated by the Image J software (Image J 1.8.0, National Institutes of Health, Bethesda, MD, USA). β-actin was used as a loading control.

### 4.8. Statistical Analysis

All experiments were performed in triplicate. Data were expressed as mean ± SD and analyzed by SPSS (SPSS 13.0 for Windows; SPSS, Inc., Chicago, IL, USA). *p* < 0.05 was regarded as significant.

## 5. Conclusions

**Figure 6:**
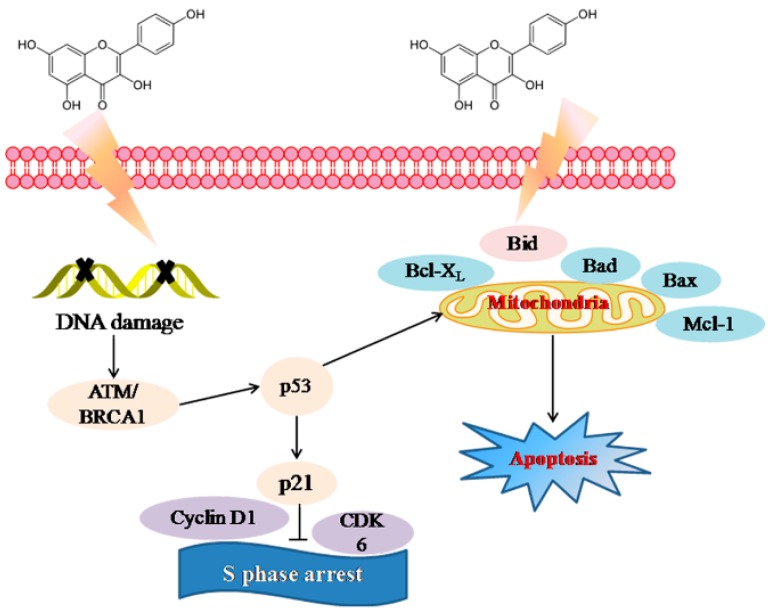
Proposed signaling pathway triggered by kaempferol in EJ cells.

In summary, kaempferol could exert remarkable anti-oxidant activity through down-regulated MDA and ROS and up-regulated SOD and GPx activities in human erythrocytes. Kaempferol also showed antiproliferative activity on bladder cancer EJ cells by inducing apoptosis accompanied with S phase arrest through activating p53 signal pathway, as shown in Figure 6. Therefore, kaempferol exhibited highly antioxidant and antiproliferative activity on bladder cancer cells EJ. All these results provided scientific validations of kaempferol bioactivities. This study showed the public that they could prevent much oxidative injury and treat bladder cancer by simply developing healthy eating habits.

## Figures and Tables

**Table 1:** Kaempferol suppresses AAPH-induced erythrocyte hemolysis.

Group	Final Concentration	Hemolysis Inhibition Rate (%)
VC (positive control)	80 μM	96.84 ± 0.0010 ^a^
Kaempferol	20 μM	81.00 ± 0.3270 ^b^
Kaempferol	40 μM	93.63 ± 0.0180 ^a^
Kaempferol	80 μM	94.80 ± 0.0058 ^a^
Damaging group (AAPH)	200 mM	42.15 ± 0.0025 ^c^

All data are expressed as means ± SD of triplicates. Means with different letters (a, b, c) are significantly different at *p* < 0.05 level.

## Abbreviations

MDA: malonaldehyde; ROS: reactive oxygen species; SOD: superoxide dismutase; GPx: glutathione peroxidase antioxidant activity; AAPH: 2′-azobis (2-amidinopropane) dihydrochloride; MTT: thiazolyl blue tetrazolium bromide; PI: propidium iodide; BCA: bicinchoninic acid; DCFH-DA: 2,7-dichlorodi-hydrofluorescein diacetate.

## References

[1] Garg M. (2015). Urothelial cancer stem cells and epithelial plasticity: Current concepts and therapeutic implications in bladder cancer. Cancer Metastasis Rev..

[2] Kasala E.R., Bodduluru L.N., Madana R.M., Athira K.V., Gogoi R., Barua C.C. (2015). Chemopreventive and therapeutic potential of chrysin in cancer: Mechanistic perspectives. Toxicol. Lett..

[3] Chen A.Y., Chen Y.C. (2013). A review of the dietary flavonoid, kaempferol on human health and cancer chemoprevention. Food Chem..

[4] Wang Y., Zhang G., Pan J., Gong D. (2015). Novel insights into the inhibitory mechanism of kaempferol on xanthine oxidase. J. Agric. Food Chem..

[5] Yao S., Wang X., Li C., Zhao T., Hai J., Fang W. (2016). Kaempferol inhibits cell proliferation and glycolysis in esophagus squamous cell carcinoma via targeting EGFR signaling pathway. Tumour Biol..

[6] Devi K.P., Malar D.S., Nabavi S.F., Sureda A., Xiao J., Nabavi S.M., Daglia M. (2015). Kaempferol and inflammation: From chemistry to medicine. Pharmacol. Res..

[7] Saw C.L.L., Guo Y., Yang A.Y., Paredes-Gonzalez X., Ramirez C., Pung D., Kong A.N.T. (2014). The berry constituents quercetin, kaempferol, and pterostilbene synergistically attenuate reactive oxygen species: Involvement of the Nrf2-ARE signaling pathway. Food Chem. Toxicol..

[8] Nothlings U., Murphy S.P., Wilkens L.R., Henderson B.E., Kolonel L.N. (2007). Flavonols and pancreatic cancer risk: The multiethnic cohort study. Am. J. Epidemiol..

[9] Cui Y., Morgenstern H., Greenland S., Tashkin D.P., Mao J.T., Cai L., Cozen W., Mack T.M., Lu Q.Y., Zhang Z.F. (2008). Dietary flavonoid intake and lung cancer—A population-based case-control study. Cancer.

[10] Dang Q., Song W., Xu D., Ma Y., Li F., Zeng J., Zhu G., Wang X., Chang L.S., He D. (2015). Kaempferol suppresses bladder cancer tumor growth by inhibiting cell proliferation and inducing apoptosis. Mol. Carcinog..

[11] Ferder L., Inserra F., Martínezmaldonado M. (2006). Inflammation and the metabolic syndrome: Role of angiotensin II and oxidative stress. Curr. Hypertens. Rep..

[12] Cimen M.Y. (2008). Free radical metabolism in human erythrocytes. Clin. Chim. Acta.

[13] Liao K.H., Lin Y.S., Macosko C.W., Haynes C.L. (2011). Cytotoxicity of graphene oxide and graphene in human erythrocytes and skin fibroblasts. ACS Appl. Mater. Interfaces.

[14] Dumaswala U.J., Zhuo L., Jacobsen D.W., Jain S.K., Sukalski K.A. (1999). Protein and lipid oxidation of banked human erythrocytes: Role of glutathione. Free Radic. Biol. Med..

[15] Miele L., Gabrieli M.L., Forgione A., Vero V., Gallo A., Capristo E., Gasbarrini G., Grieco A. (2006). Oxidative stress in metabolic syndrome and nonalcoholic steatohepatitis. Is it possible a role for vitamins in clinical practice?. Recenti Prog. Med..

[16] Abraham A.G., O’Neill E. (2014). PI3K/Akt-mediated regulation of p53 in cancer. Biochem. Soc. Trans..

[17] Jin Y., Zhang S., Tao R., Huang J., He X., Qu L., Fu Z. (2014). Oral exposure of mice to cadmium (II), chromium (VI) and their mixture induce oxidative- and endoplasmic reticulum-stress mediated apoptosis in the livers. Environ. Toxicol..

[18] Alvarez-Suarez J.M., Dekanski D., Ristić S., Radonjić N.V., Petronijević N.D., Giampieri F., Astolfi P., González-Paramás A.M., Santos-Buelga C., Tulipani S. (2011). Strawberry polyphenols attenuate ethanol-induced gastric lesions in rats by activation of antioxidant enzymes and attenuation of MDA increase. PLoS ONE.

[19] Fan C., Jiang J., Yin X., Wong K.H., Zheng W., Chen T. (2012). Purification of selenium-containing allophycocyanin from selenium-enriched *Spirulina platensis* and its hepatoprotective effect against t-BOOH-induced apoptosis. Food Chem..

[20] Liao W., Chen L., Ma X., Jiao R., Li X., Wang Y. (2016). Protective effects of kaempferol against reactive oxygen species-induced hemolysis and its antiproliferative activity on human cancer cells. Eur. J. Med. Chem..

[21] Filomeni G., Desideri E., Cardaci S., Graziani I., Piccirillo S., Rotilio G., Ciriolo M.R. (2010). Carcinoma cells activate AMP-activated protein kinase-dependent autophagy as survival response to kaempferol-mediated energetic impairment. Autophagy.

[22] Choi E.M. (2011). Kaempferol protects MC3T3-E1 cells through antioxidant effect and regulation of mitochondrial function. Food Chem. Toxicol..

[23] Vellosa J.C.R., Regasini L.O., Khalil N.M., da Silva Bolzani V., Khalil O.A.K., Manente F.A., Netto H.P., de Faria Oliveira O.M.M. (2011). Antioxidant and cytotoxic studies for kaempferol, quercetin and isoquercitrin. Eclética Química.

[24] Zhang T.T., Lu C.L., Jiang J.G. (2015). Antioxidant and anti-tumour evaluation of compounds identified from fruit of *Amomum tsaoko* Crevost et Lemaire. J. Funct. Foods..

[25] Qiu W., Lin J., Zhu Y., Zhang J., Zeng L., Su M., Tian Y. (2017). Kaempferol modulates DNA methylation and downregulates DNMT3B in bladder cancer. Cell. Physiol. Biochem..

[26] Kumari S., Mehta S.L., Li P.A. (2012). Glutamate induces mitochondrial dynamic imbalance and autophagy activation: Preventive effects of selenium. PLoS ONE.

[27] Li S., Pu X.P. (2011). Neuroprotective effect of kaempferol against a 1-methyl-4-phenyl-1,2,3,6-tetrahydropyridine-induced mouse model of Parkinson’s disease. Biol. Pharm. Bull..

[28] Luo X., Yu X., Liu S., Deng Q., Liu X., Peng S., Li H., Liu J., Cao Y. (2015). The role of targeting kinase activity by natural products in cancer chemoprevention and chemotherapy. Oncol. Rep..

[29] Zhao L., Su J., Li L., Chen J., Hu S., Zhang X., Chen T. (2014). Mechanistic elucidation of apoptosis and cell cycle arrest induced by 5-hydroxymethylfurfural, the important role of ROS-mediated signaling pathways. Food Res. Int..

[30] Wu P., Liu S., Su J., Chen J., Li L., Zhang R., Chen T. (2017). Apoptosis triggered by isoquercitrin in bladder cancer cells by activating the AMPK-activated protein kinase pathway. Food Funct..

[31] Peramaiyan R., Thamaraiselvan R., Natarajan N., Rajendran P., Yutaka N., Ikuo N. (2014). Kaempferol, a potential cytostatic and cure for inflammatory disorders. Eur. J. Med. Chem..

[32] Meng X., Dong X., Wang W., Yang L., Zhang X., Li Y., Chen T., Ma H., Qi D., Su J. (2018). Natural borneol enhances paclitaxel-induced apoptosis of ESCC cells by inactivation of the PI3K/AKT. J. Food Sci..

[33] Xie F., Su M., Qiu W., Zhang M., Guo Z., Su B., Liu J., Li X., Zhou L. (2013). Kaempferol promotes apoptosis in human bladder cancer cells by inducing the tumor suppressor, PTEN. Int. J. Mol. Sci..

[34] Barve A., Chen C., Hebbar V., Desiderio J., Saw L.L., Kong A.N. (2010). Metabolism, oral bioavailability and pharmacokinetics of chemopreventive kaempferol in rats. Biopharm. Drug Dispos..

[35] Rajbhandari R., Peng N., Moore R., Arabshahi A., Wyss J.M., Barnes S., Prasain J.K. (2011). Determination of cranberry phenolic metabolites in rats by liquid chromatography-tandem mass spectrometry. J. Agric. Food Chem..

[36] Zhao L., Chen J., Su J., Lin L., Hu S.Q., Li B., Zhang X., Xu Z., Chen T. (2013). In vitro antioxidant and antiproliferative activities of 5-Hydroxymethylfurfural. J. Agric. Food Chem..

[37] Tang X., Rooij M.D., Jong L.D. (2007). Volume change measurements of rice by environmental scanning electron microscopy and stereoscopy. Scanning.

[38] Lee J.S., Hong E.K. (2010). Hericium erinaceus enhances doxorubicin-induced apoptosis in human hepatocellular carcinoma cells. Cancer Lett..

[39] Chen J., Li L., Su J., Li B., Zhang X., Chen T. (2015). Proteomic analysis of G2/M arrest triggered by natural borneol/curcumin in HepG2 cells, the importance of ROS-p53 pathway. J. Agric. Food Chem..

[40] Chen J., Li L., Su J., Chen T. (2014). Natural borneol enhances bisdemethoxycurcumin-induced cell cycle arrest in the G2/M phase through up-regulation of intracellular ROS in HepG2 cells. Food Funct..

